# Role of nonhuman primate models in the discovery and clinical development of selective progesterone receptor modulators (SPRMs)

**DOI:** 10.1186/1477-7827-4-S1-S8

**Published:** 2006-10-09

**Authors:** Kristof Chwalisz, Ramesh Garg, Robert Brenner, Ov Slayden, Craig Winkel, Walter Elger

**Affiliations:** 1 TAP Pharmaceutical Products Inc., Lake Forest, Illinois, USA; 2 Oregon Regional Primate Research Center, Oregon Health Sciences University, Beaverton, OR 97006, USA; 3 EnTec GmbH, Hamburg and Jena, Germany; 4 Schorlemerallee 12B, 14195 Berlin-Dahlem, Germany

## Abstract

Selective progesterone receptor modulators (SPRMs) represent a new class of progesterone receptor ligands that exert clinically relevant tissue-selective progesterone agonist, antagonist, partial, or mixed agonist/antagonist effects on various progesterone target tissues in an in vivo situation depending on the biological action studied. The SPRM asoprisnil is being studied in women with symptomatic uterine leiomyomata and endometriosis. Asoprisnil shows a high degree of uterine selectivity as compared to effects on ovulation or ovarian hormone secretion in humans. It induces amenorrhea and decreases leiomyoma volume in a dose-dependent manner in the presence of follicular phase estrogen concentrations. It also has endometrial antiproliferative effects. In pregnant animals, the myometrial, i.e. labor-inducing, effects of asoprisnil are blunted or absent. Studies in non-human primates played a key role during the preclinical development of selective progesterone receptor modulators. These studies provided the first evidence of uterus-selective effects of asoprisnil and structurally related compounds, and the rationale for clinical development of asoprisnil.

## Background

Progesterone plays a crucial role in controlling various reproductive functions. It is the natural ligand of the progesterone receptor (PR), which is expressed in various tissues in the body, predominantly in the reproductive tract. The isolation of progesterone in 1934 [1-3] led to the search for synthetic, orally active progestins (PR agonists) that have found broad applications in fertility control and hormone therapy. Since the discovery of mifepristone in 1981 by the scientists of Roussel Uclaf [4], several progesterone antagonists (PAs) became available for preclinical and clinical evaluation [5]. More recently, selective progesterone receptor modulators (SPRMs) have been synthesized and biologically characterized. SPRMs represent a new class of PR ligands that exert clinically relevant tissue-selective progesterone agonist, antagonist, partial, or mixed agonist/antagonist effects on various progesterone target tissues in an *in vivo* situation depending on the biological action studied [6]. Asoprisnil (J867), a novel steroidal compound that belongs to the class of 11β-benzaldoxime-substituted estratrienes [7,8], is being studied in women with symptomatic uterine leiomyomata and endometriosis. It shows high PR specificity, mixed PR agonist/antagonist activity, and high degree of uterine selectivity in animal models and humans [8]. Unlike PAs, asoprisnil does not induce labor in relevant models of pregnancy and parturition.

Studies in cynomolgus monkeys provided the first evidence that the 11β-benzaldoxime-substituted SPRMs may induce amenorrhea by directly targeting the endometrium and have direct endometrial antiproliferative effects, irrespective of ongoing ovulatory cycles [9]. The endometrial effects of SPRMs and PAs are specific to menstruating primates (Old World monkeys, humans) since variable effects were observed in non-menstruating species (rodents, rabbits, tree shrews, and New World monkeys) [9]. The endometrial antiproliferative effects and the endometrium specific vascular effects were subsequently confirmed in women treated with asoprisnil [10]. Human studies also showed that asoprisnil improved the symptoms of leiomyomata, reduced leiomyoma volume [11], and reduced pain associated with endometriosis [12]. In this brief review, we discuss the discovery and early clinical development of asoprisnil and related SPRMs, focusing on the effects of these compounds on the primate uterus.

## Discovery and early development of 11β-benzaldoxime-substituted SPRMs

**Figure 1:**
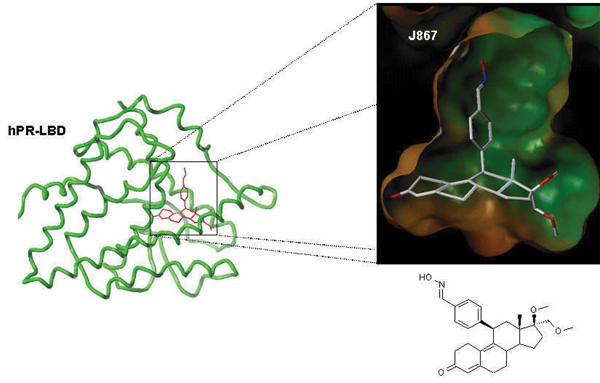
**Asoprisnil in the binding domain of the progesterone receptor**.

**Figure 2:**
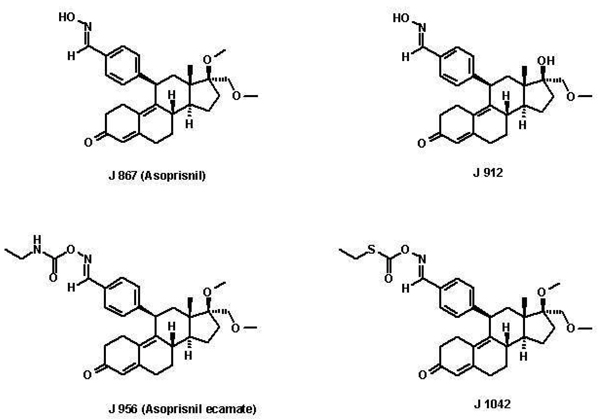
**Chemical structures of major 11β-benzaldoxime substituted SPRMs**.

**Figure 3:**
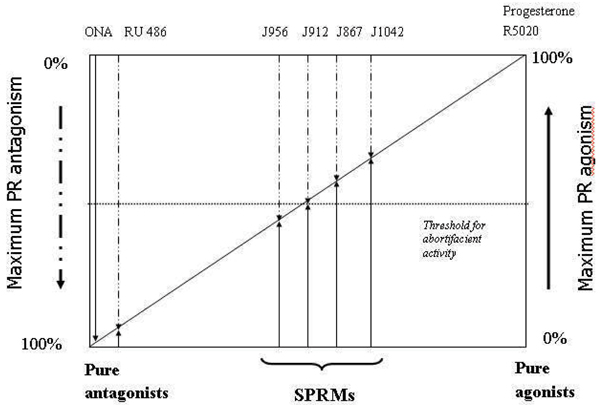
**Spectrum of progesterone agonist and antagonist activities of major 11β-benzoxime-substituted SPRMs and PAs**. The figure is based on studies in rabbits (McPhail test) and guinea pigs (luteolysis inhibition assay, induction of labor). ONA, onapristone; R520, pure progestin promegestone; J867, asoprisnil; PR, progesterone receptor. From [54] with permission.

Asoprisnil and structurally related SPRMs (Figures 1 and 2) were identified at EnTec GmbH and Jenapharm GmbH (Jena, Germany) during a SPRM ('mesoprogestin') drug discovery program [13]. The aim of this program was to find PR ligands with mixed PR agonist/antagonist, antiproliferative effects on the endometrium, no antiglucocorticoid effects, and weak or absent abortifacient activity for treatment of gynecological disorders. The screening program included a range of receptor binding studies and a hierarchy of in vivo tests. The selection of candidate compounds was performed primarily in animal models, including non-pregnant and pregnant guinea pigs, rabbits, rats, and monkeys. The assessment of endometrial effects was of crucial importance for the final selection of candidate compounds. This assessment was performed in cynomolgus and rhesus macaques because the endometrium of these monkeys shows striking similarities with that of women with respect to hormonal regulation and morphological changes during the menstrual cycle [14]. Based on the data from animal studies generated during this program, it was possible to rank the new SPRMs and the reference compounds according to the presence and absence of partial PR agonist and antagonist activities (Figure 3). Compounds with high PR agonist activity had low abortifacient activity and high ability to suppress estrogen effects in the uterus [13]. Asoprisnil (J867) was selected for further development based on its pronounced PR agonist and endometrial antiproliferative effects and the absence of labor-inducing activity.

## Effect of SPRMs on the primate endometrium

The primate endometrium is a highly specialized tissue composed of different cell components, including luminal and glandular epithelium, endometrial stroma, lymphoid and non-lymphoid cells, and blood vessels [15]. The endometrium is probably the most dynamic tissue in the human body. In humans and menstruating ("old-world") non-human primates the endometrium undergoes cyclic changes characterized by regeneration and proliferation during the follicular phase of the ovarian cycle, secretory differentiation during the luteal phase, and menstruation accompanied by vasoconstriction of spiral arteries and changes in extracellullar matrix that occur after the physiologic decline in progesterone concentrations that occur at the end of the luteal phase. Spiral arteries are key vessels that control menstruation [16]. These vessels are unique to the primate endometrium and are highly sensitive to progesterone. They seem to be controlled by perivascular stromal cells (pericytes), which show very high PR density [17].

Estrogen is the primary mitogen in the endometrium, acting primarily on the epithelium. "Unopposed" estrogen treatment (without a progestin phase) leads to proliferation of glandular and stromal cells and may lead to endometrial hyperplasia and possibly endometrial cancer. Endometrial hyperplasia is characterized by increased proliferation of the endometrial glandular epithelium, resulting in an increase of the gland/stroma ratio. Progesterone and synthetic progestins oppose the effects of estrogen on the epithelium, and thereby protect the endometrium from the development of hyperplasia. The specific mechanisms that have been proposed to explain the antiproliferative action of progesterone on the uterine epithelium include: (i) down-regulation of estrogen receptors (ER) in target tissues, (ii) induction of the endometrial enzyme 17β-hydroxysteroid dehydrogenase Type 2 that catalyzes the conversion of estradiol to the less active estrone, (iii) reduction in estrogen-induced specific protein expression, and (iv) inhibition of estrogen-induced proto-oncogenes (cjun, cfos), which act as growth factors in the endometrium (reviewed by [9]). Although all the mechanisms seem to be involved, the down-regulation of ER in target tissues most likely plays the primary role in this respect.

During the drug discovery program, we conducted studies in cynomolgus monkeys to determine the effects of selected SPRMs in the primate endometrium [13]. One of our initial studies compared the endometrial effects of a "model" SPRM with high agonist activity (J1042) with those of PAs (ZK 137 316 and ZK 230 211). This study showed that all three compounds have a profound antiproliferative effect on the endometrium characterized by a decrease in endometrial thickness and reduction in mitotic activity in both endometrial epithelium and stroma. Endometrial stroma appeared compact in all treatment groups, which suggested a progesterone antagonist effect of J1042 and the reference PAs [9,18]. However, J1042, but not the reference PAs, induced secretory activity of the endometrial glands characterized by glandular sacculation with subnuclear vacuolization and secretion, suggesting a weak progesterone agonistic effect. In contrast, both PAs produced degenerative changes in endometrial glands without any secretory changes. In addition, morphometric studies revealed that J1042 reduced intraluminal diameter of spiral arteries in the basalis to a higher degree than the PAs, but did not cause hyalinizing degeneration of spiral arteries as had previously been observed in animals treated with PAs. This study, which showed for the first time the presence of partial secretory effects of a SPRM in the primate endometrium, played a key role in the conceptualization and discovery of tissue selectivity of SPRMs.

**Figure 4:**
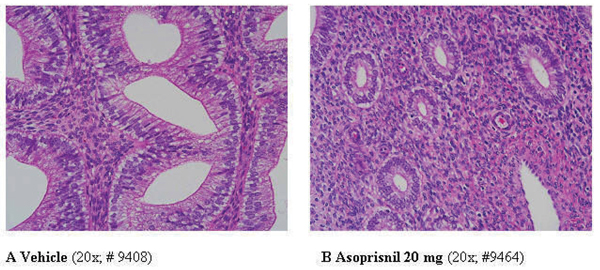
**The effects of asoprisnil on endometrial morphology in female cynomolgus monkeys (39-week toxicity study in adult cynomolgus monkeys)**. Photomicrographic comparison of glandular morphology in a control monkey during the secretory phase with an animal treated with asoprisnil at a dose of 20 mg/kg. Asoprisnil induced glandular atrophy. Magnification 20×. From [8] with permission from Elsevier.

Chronic toxicological studies conducted in intact cynomolgus monkeys of 39-week duration consistently showed endometrial antiproliferative effects of asoprisnil [8] and asoprisnil ecamate (J956) (unpublished data). In the 39-week toxicologic study, asoprisnil was administered orally once daily to intact cynomolgus monkeys at doses of 20, 60, 160 and 480 mg/kg/day. At these dosages, the systemic drug exposure in female monkeys as area under the plasma concentration curves (AUCs) combined for the parent drug and J912, a major metabolite, were 1116, 5345, 16816 and 28838 ng•hr/mL, respectively. Compared to the control animals, this treatment induced amenorrhea and profound endometrial atrophy (early proliferative stage) that was accompanied by a trend towards more compact stromal appearance, without any evidence of secretory changes in endometrial glands (Figure 4). This effect was observed in females of all asoprisnil-treated groups (n = 4–5) except for females of the highest dose group where the effect was observed in 4 of the 5 females. Menstrual activity was suppressed in all asoprisnil-treated females within 2 weeks after the initiation of treatment. Mean estradiol serum concentrations were at early follicular levels.

The effect of asoprisnil on endometrial morphology was consequently studied in more detail in cynomolgus monkeys treated with lower doses of asoprisnil for a shorter period of time [19]. A total of 24 ovarian-intact, cycling animals were administered either saline (n = 6) or vehicle (n = 4) as controls, or three doses of asoprisnil (10 mg/kg [n = 4], 30 mg/kg [n = 4] and 90 mg/kg [n = 6]) orally, once daily for 90 days. Four animals from each group were sacrificed at the end of treatment and two animals from each of the saline and 90 mg/kg groups were held without treatment for a recovery period of 28 days after which they were also sacrificed. In this study, asoprisnil was suspended in a vehicle containing 10% ethanol, 35% PEG (polyethylene glycol) 300 and 55% Cremophor-EL (polyoxyethylenglyceroltriricinoleat 35, polyoxyl 35 castor oil) to enhance its oral absorption. The AUCs at these dosages in female monkeys were 368, 2495 and 13422 ng•hr/mL, respectively. The entire reproductive tract was removed from all animals and assessed for general histological changes, including spiral artery development, endometrial thickness, and stromal compaction. Endometrial thickness was assessed by measuring the distance from the luminal surface to the myometrial border, and stromal compaction was quantified by computerized assessment of the number of stromal cells per unit area. The effects on proliferative state were quantified by assessing two markers of proliferation, Ki-67 and phosphorylated histone 3 (Phospho H3). Ki-67 is a nuclear protein expressed during all stages of the cell cycle except G0 and is a standard index of overall cellular proliferation. Because histone 3 is phosphorylated only during mitosis and is expressed only in mitotic chromosomes, Phospho H3 staining provides a direct indication of mitotic activity[20]. The effects of asoprisnil on ovarian, cervical and vaginal histology were also assessed.

In this study, the ovaries in the saline and vehicle controls were all normal in appearance, marked by either antral follicles or functional corpora lutea. The endometria in all control animals were either in the proliferative or secretory phases. However, in the asoprisnil-treated animals, the two higher doses suppressed ovulation and progesterone secretion. The previous 39-week toxicological study showed that estradiol was maintained at follicular phase levels, and in the current study, the vagina was in an estrogenized state (assessed by degree of cornification) in all asoprisnil-treated animals, indicating that (i) estradiol levels were physiologically adequate, and (ii) asoprisnil did not block estrogen action in the vagina.

**Figure 5:**
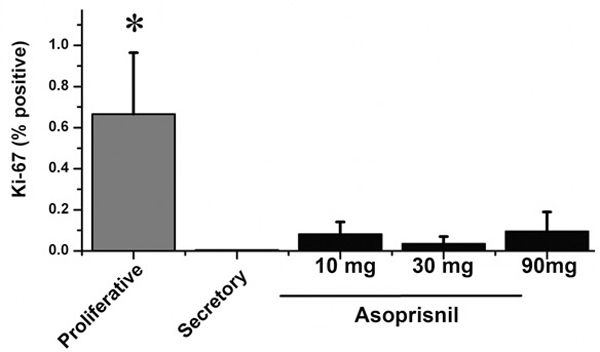
**Asoprisnil suppresses the Ki-67 index in cynomolgus macaque endometrium**. All doses of asoprisnil inhibited endometrial Ki-67 index compared to control animals in the proliferative phase of the cycle. Bars marked with an asterisk are significantly different from all other bars (p < 0.05).

**Figure 6:**
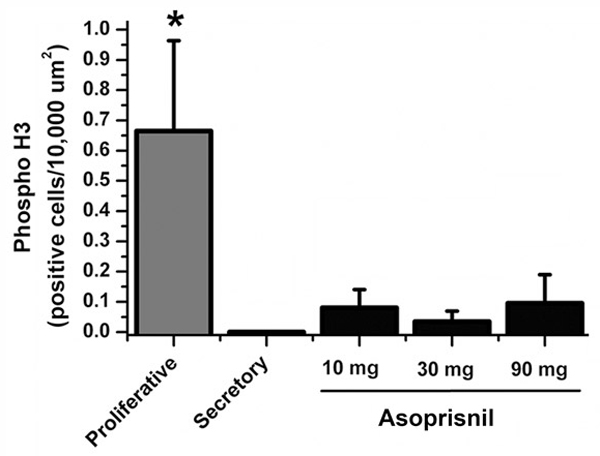
**Asoprisnil suppresses the Phospho H3 index in cynomolgus macaque endometrium**. All doses of asoprisnil inhibited endometrial Phospho-H3 index compared to control animals in the proliferative phase of the cycle. Bars marked with an asterisk are significantly different from all other bars (p < 0.05).

**Figure 7:**
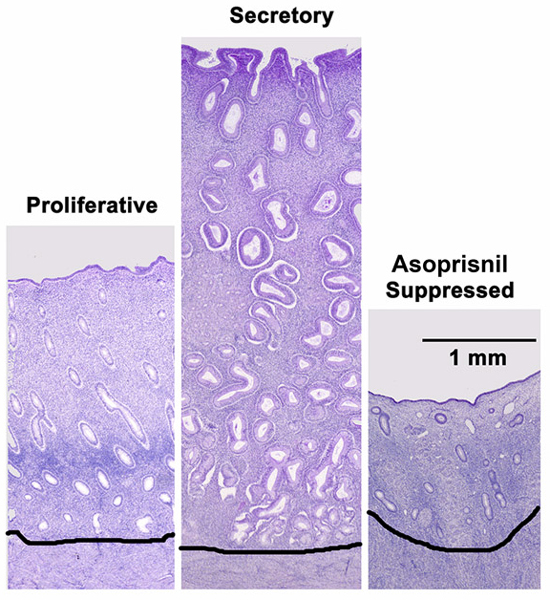
**Effect of asoprisnil on overall endometrial histology**. Photomicrographic comparison of endometrial histology of typical control animals (proliferative and secretory phases) versus asoprisnil-treated (90 mg/kg) animals. Original magnification ~4×.

**Figure 8:**
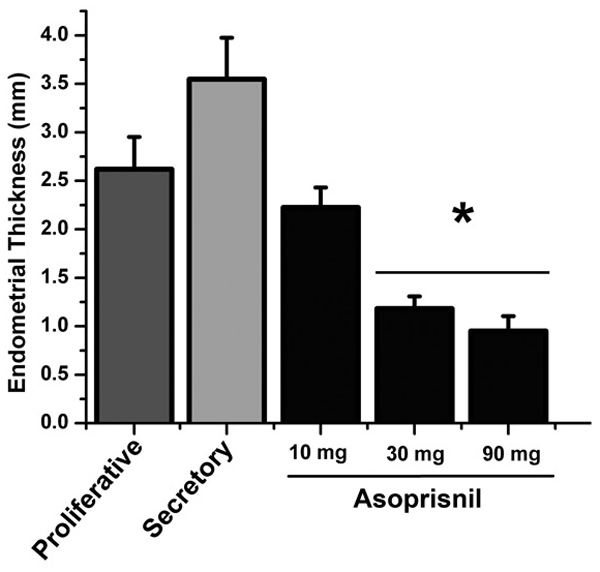
**Effects of asoprisnil on endometrial thickness**. The two higher doses of asoprisnil significantly inhibited endometrial thickness compared to all other groups. Bars marked with an asterisk are significantly different from all other bars (p < 0.05).

However, all doses of asoprisnil significantly suppressed the proliferation markers Ki-67 (Figure 5) and Phospho-H3 (Figure 6) in the endometrial glands, and the two higher doses caused significant shrinkage in endometrial thickness (Figures 7, 8) without inducing progestational effects such as glandular sacculation and secretion. There was a trend for stromal compaction, but no evidence of spiral artery degeneration. In sum, asoprisnil had an endometrial antiproliferative effect, but unlike a PA, did not induce any severe stromal compaction or spiral artery degeneration.

In sum, asoprisnil had reversible antiovulatory and endometrial antiproliferative effects, but did not suppress estrogen action in other parts of the reproductive tract of ovarian-intact cynomolgus macaques.

## Effects of asoprisnil on the human uterus

Phase I and Phase II trials confirmed tissue-selective effects of asoprisnil in the human uterus. Some of the clinical studies with asoprisnil have been published [10], or reported in abstract form [11]; [21]; [12].

In a Phase I study, the effects of asoprisnil versus placebo were evaluated in 60 healthy regularly cycling premenopausal women during treatment for 28 days at daily oral doses ranging from 5 mg to 100 mg/day, starting during the first two days of the menstrual cycle [10]. Asoprisnil consistently prolonged the menstrual cycle at doses ≥ 10 mg/day. However, the effects on luteal phase progesterone indicative of luteinization were inconsistent and lacked dose dependency. The estradiol levels of women treated with asoprisnil were within the range of the follicular phase. This study demonstrated that asoprisnil suppresses menstruation by primarily targeting the endometrium.

In a Phase II setting, the effects of asoprisnil 5 mg, 10 mg, and 25 mg on bleeding patterns and various leiomyoma endpoints were evaluated in a double-blind, placebo-controlled study of patients with uterine leiomyomata [11]. The patients were treated for 3 months. Asoprisnil significantly suppressed both the duration and intensity of uterine bleeding in a dose-dependent manner as evidenced by bleeding diaries, and high amenorrhea rates (0%, 28.1%, 64.3%, and 83.3% at placebo, 5 mg, 10 mg and 25 mg, respectively). There was a significant increase in hemoglobin concentrations by week 12 in all asoprisnil groups compared to placebo. Asoprisnil also significantly reduced menorrhagia scores in patients with leiomyomata [21]. The suppressive effects of asoprisnil on endometrial bleeding were evident within the first month and maintained throughout the entire treatment period. These effects were accompanied by a dose-dependent reduction in the volumes of the largest leiomyoma as measured by ultrasound. Asoprisnil had no statistically significant effects on ovarian estrogens (estradiol and estrone). Consistent with the absence of antiglucocorticoid activity in humans at clinically relevant doses, there were no increases in serum concentrations of cortisol or dehydroepiandrosterone sulphate (DHEA-S) with asoprisnil [11,22].

**Figure 9:**
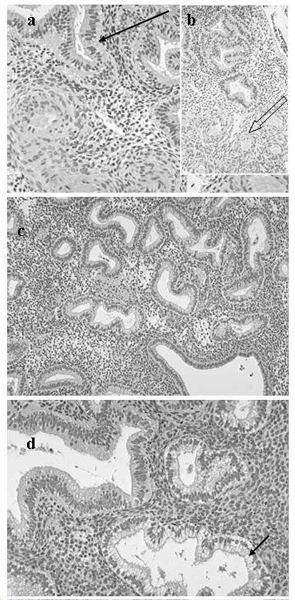
Representative endometrial biopsies of subjects treated with asoprisnil for 28 days. The photographs show examples consistent with the diagnosis "non-physiologic secretory effect". **a**: Weakly secretory glands with vacuoles below nuclei (solid arrow; Subject MN-6; asoprisnil 10 mg QD); **b**: Thick-walled vessels (open arrow; Subject MN-6; asoprisnil 10 mg); **c**: Mild to moderate secretory glands at low magnitude. Note area of relatively dense stroma. (subject MN, 25 mg BID); **d**: Moderate secretory glands with subnuclear vacuoles at higher magnitude (solid arrow; subject MN, 25 mg BID). QD, once a day. BID, twice a day. From [10] with permission.

In Phase I and II studies, the endometrial biopsies obtained from women treated with asoprisnil studies showed asynchronous differentiation of endometrial epithelium and stroma consistent with its mixed progesterone agonist/antagonist activity. These changes have not been previously observed with any other hormonal treatment. Furthermore, these biopsies could not be assessed using the Noyes criteria of endometrial dating [23], as originally planned. Therefore, an expert panel of gynecological pathologists in collaboration with Diagnostic Cytology Laboratories, Indianapolis, IN, developed two new diagnostic categories describing specific effects of asoprisnil on the endometrium. The first category, "non-physiologic secretory effect," is characterized by weak secretory effects on endometrial glands without any mitotic figures and variable effects on endometrial stroma ranging from stromal compaction to focal predecidual changes. The second category, "secretory pattern, mixed type," differs from the first category by the presence of isolated mitotic figures in endometrial glands. These appearances were already evident after asoprisnil treatment for 28 days at doses of 5 mg and higher, and became more frequent after treatment for 3 months [24]. The increase in the frequency of non-physiologic secretory patterns was accompanied with a decrease in proliferative patterns [10]. Occasionally, dilated glands filled with secretory material and covered by inactive epithelium were observed with no evidence of hyperplastic changes [24]. Interestingly, clusters of unusual "thick-walled" arterial vessels were consistently found in the endometrium of women treated with asoprisnil (Figure 9). This effect is specific to the endometrium since no changes in the wall of arterial vessels were found in non-endometrial tissues of the reproductive tract [24].

In summary, asoprisnil demonstrated antiproliferative effects on the endometrium in the presence of follicular phase estradiol concentrations in both humans and monkeys. However, these studies also revealed some differences between the non-human primate models and humans with respect to endometrial effects. In the human endometrium, asoprisnil treatment for up to 3 months was associated with non-physiologic secretory effects in the glandular epithelium and presence of unusual "thick-walled" arterial vessels in the stroma [24]. In cynomolgus monkeys neither secretory changes in endometrial glands, nor formation of thick-walled endometrial spiral arteries were observed, pointing out to some important differences in the steroid receptor pharmacology of the monkey and human endometrium. The reason for these differences is unclear. However, our studies with asoprisnil and previous experience with PAs suggest that the macaque endometrium might be more sensitive to PR-ligands than the human endometrium, which would explain more dramatic effects of SPRMs and PAs in these animals. Therefore, in spite of many similiarities between monkey and human endometrium, caution is warranted in extrapolating the data generated in nonhuman primates to humans.

## Effects of SPRMs on the breast tissue in cynomolgus monkeys

The potential effects of SPRMs on the breast tissue are of special interest. In primates, mammary gland development is controlled by a complex interplay between the reproductive hormones (estradiol, progesterone, and prolactin), and local growth factors [25]. There is growing evidence that progesterone is an important mitogen in epithelial breast cells [26]. On the one hand, PAs suppress mammary gland proliferation and inhibit growth of PR-positive mammary gland tumors in animal models [27]. On the other hand, mitotic activity in normal breast tissue peaks during the luteal phase, and synthetic progestins consistently increase mammographic breast density, which is a surrogate parameter of breast proliferation [28,29], as well as upregulate the expression of proliferation markers in the mammary gland [30].

Inhibitory effects of asoprisnil on mammary gland development were consistently observed in a 39-week toxicological study in intact female cynomolgus monkeys [31]. Although these effects are consistent with antiproliferative effects of asoprisnil on mammary gland proliferation, they should be interpreted with caution because of high doses used in toxicological studies, and potential differences between macaques and humans regarding hormonal regulation of breast proliferation and differentiation.

## Mechanism of tissue selectivity of SPRMs

Our studies in non-human primates and humans reviewed above revealed that asoprisnil and other 11β-benzaldoxime-substituted SPRMs exert tissue-selective effects on the uterus in the presence of follicular phase estrogen concentrations and no antiglucocorticoid effects. These compounds can abolish endometrial bleeding and suppress endometrial proliferation in both macaques and humans. Inhibitory effects of leiomyoma growth were observed in human studies with asoprisnil. Our studies show that asoprisnil exhibits inhibitory effects on (i) endometrial bleeding, (ii) endometrial proliferation, and (iii) leiomyoma volume in humans, predominantly via tissue-specific (uterine) mechanisms. These effects, discussed in more detail below, are likely to reflect target tissue specific manifestation of PR modulation that affects downstream pathways of PR, ER AR and most likely other, still unidentified pathways.

### Control of endometrial bleeding

Amenorrhea was consistently observed in asoprisnil-treated cynomolgus monkeys [8]. In women, amenorrhea was already observed within 30 days of commencement of asoprisnil treatment, irrespective of the effect of luteinization suggestive of ovulation. This effect is sustained during longer treatment, with minimal bleeding abnormalities such as spotting and breakthrough bleeding [10,11]. These observations suggest that the endometrial arteries are stable during treatment with asoprisnil.

Although the mechanism of asoprisnil-induced amenorrhea is still not completely understood, the current evidence suggests that asoprisnil may have a direct or indirect inhibitory effect on endometrial spiral arteries. Interestingly, asoprisnil-induced morphological changes in spiral arteries, characterized by thickening of the wall, clearly differ from those commonly observed in women using long-acting progestins or levonorgestrel-containing intrauterine systems. These treatments are associated with the formation of "thin-walled" microvessels that are very fragile, and frequently lead to breakthrough bleeding [32-34]. Our working hypothesis is that asoprisnil controls endometrial bleeding by targeting the perivascular cells in the endometrium, which are characterized by a high density of PRs [17]. This hypothesis, which is currently under investigation, would explain tissue-specific effects of asoprisnil on endometrial vasculature.

### Endometrial antiproliferative effect

Asoprisnil and structurally related SPRMs have endometrial antiproliferative effects in macaques and humans. The exact mechanism of this effect is still not fully understood. The effects of asoprisnil on the human endometrium are currently being investigated using various morphological, immunohistochemical and molecular endpoints. These studies should provide more insight into the molecular mechanisms underlying this effect, in particular the role of the partial progesterone agonist and antagonist activities of asoprisnil in the endometrium. Based on experiments conducted in cynomolgus monkeys, we previously proposed that the endometrial antiproliferative effect of SPRM and PAs might be due to the reduction in endometrial blood flow as consequence of vascular changes in the endometrium [9]. However, this hypothesis has not yet been confirmed in humans. More recently the role of endometrial AR has been emphasized as a potential mechanism of the endometrial antiproliferative effects of PAs [35-38]. Androgens are known to inhibit estrogen effects in the primate endometrium [38]. The AR hypothesis was proposed based on the observation of greatly enhanced expression of AR in the endometrial glands of macaques treated with various PAs and in women treated with mifepristone [35]. Furthermore, the AR antagonist flutamide reversed the endometrial antiproliferative effect of the PA as evidenced by changes in mitotic index, endometrial height and weight [39]. Since up-regulation of AR was observed in the endometrial stroma of cynomolgus monkeys treated with asoprisnil, its antiproliferative effect on endometrial glandular epithelium might be mediated by AR-mediated stromal growth factors [19]. In addition, asoprisnil has weak androgenic properties, which may play a role in this respect [8].

### Reduction in leiomyoma volume

Several mechanisms, which are under investigation, might be involved in asoprisnil-induced reduction in leiomyoma volume, including a direct, PR-mediated antiproliferative and pro-apoptotic effects on leiomyoma cells, inhibition of growth factors, modulation of extracellullar matrix synthesis, reduction in uterine blood flow, etc.

The results of an in vitro study with leiomyoma and myometrial primary cell cultures showed that asoprisnil inhibits proliferation and induces apoptosis of leiomyoma cells without having any effects on the normal myometrial cells [40]. In this study, the proliferation marker PCNA was used to quantify the effects on proliferation, whereas TUNEL assay was used to determine the effects on apoptosis. In a second study, asoprisnil also inhibited the expression of EGF, IGF-I, TGFβ3 and their receptors in cultured leiomyoma cells without affecting their expression in matching myometrial cells [41]. In addition, a decrease in uterine blood flow following treatment with asoprisnil may contribute to this effect. This hypothesis is currently under evaluation in clinical studies.

### Molecular basis for tissue-selective effect of SPRMs

The molecular mechanism of tissue selectivity of steroid receptor modulators has been recently proposed based on the ability of a liganded PR to interact with different coregulators depending on the specific ligand [42-45]. Coregulators (coactivators and corepressors) are nuclear proteins that form multiple complexes with nuclear receptors and modulate their transcriptional activity [46-49]. Coactivators enhance transcriptional activity of nuclear receptors, whereas corepressors elicit inhibitory effects on nuclear receptors. The PAs either favor interaction of PR with corepressors or inhibit interactions with coactivators, whereas "pure" PR agonists promote the interaction of the nuclear receptor with coactivators [50,51]. SPRMs with partial PR agonist activity induce an intermediate state of interaction between nuclear receptors and coactivators [45,50,52]. Since the availability of both coactivators and corepressors is dependent on tissue and hormonal milieu, the cell type- and promoter-specific differences in coregulator recruitment determine the tissue selectivity of SPRMs [6,45]. More recent molecular studies show that asoprisnil-liganded PR promotes the recruitment of the coactivator SRC-1 in an in vitro model [53] which could explain the partial agonist effects observed in animal models and humans. Molecular studies of coregulator expression in human tissues exposed to asoprisnil are ongoing. These studies should provide more insight into the molecular mechanism of tissue selectivity of asoprisnil in the uterus.

## Summary and Conclusion

Studies in non-human primates played a key role in the conceptualization and discovery of tissue selectivity of SPRMs. They provided the first evidence of the endometrial antiproliferative effects of asoprisnil and other 11β-benzaldoxime substituted SPRMs in the primate model, as characterized by a decrease in mitotic counts, Phospho H3 expression and endometrial thickness. These models also showed that PR modulation results in a complex interaction with ER- and AR-mediated responses. Uterus-selective effects of asoprisnil have been confirmed in clinical studies in humans. Clinical studies in healthy volunteers (Phase I) and patients with leiomyomata showed that asoprisnil induces dose-dependent inhibition of uterine bleeding predominantly due to an endometrial effect. Furthermore, our Phase II studies showed that asoprisnil reduces the volume of uterine leiomyomata in a dose-dependent manner. Although the mechanism of this effect is not fully explored, the in vitro studies with leiomyoma primary cell cultures, as well as morphological investigations of leiomyoma tissue suggest a increase in apoptosis accompanied by a decrease in proliferation after treatment with asoprisnil. These effects seem to be cell-specific, since no such changes were observed in myometrial cells.

Asoprisnil induces "non-physiologic secretory effects" on the human endometrium during treatment for up to 3 months. These effects are consistent with endometrial antiproliferative effects of asoprisnil, without evidence of hyperplastic changes. However, long-term studies with asoprisnil will be necessary to fully assess the endometrial effects of asoprisnil.

## Competing interests

Kristof Chwalisz, Ramesh Garg, and Craig Winkel are employees of TAP Pharmaceutical Products Inc. Walter Elger and Kristof Chwalisz are coinventors of several asoprisnil patents. Robert Brenner has a consulting agreement with TAP Pharmaceutical Products Inc. Ov Slayden has a consulting agreement with Schering AG.

## Authors' contributions

KC: designed most of clinical and molecular studies with asoprisnil; drafted the manuscript. RG conducted and evaluated toxicology studies in monkeys. RB conducted and interpreted primate studies. OS conducted and interpreted primate studies. WE participated in the design of animal and clinical studies.

All authors critically revised the manuscript and have approved the final version.
